# HSP90 proteins in the scenario of tumor complexity

**DOI:** 10.18632/oncotarget.16266

**Published:** 2017-03-16

**Authors:** Andrea Rasola

**Affiliations:** Department of Biomedical Sciences, University of Padova, Italy

**Keywords:** chaperone, HSP90, TRAP1, tumor metabolism, breast cancer

Molecular chaperones are sophisticated machines that make possible the optimal activity of proteins by assisting their folding, assembly in multimeric complexes, conformational changes, subcellular trafficking and degradation. Most neoplastic cells overexpress molecular chaperones in order to cope with the high risk of incorrect protein folding caused by exposure to several stress stimuli, such as nutritional and pH fluctuations, inconstant oxygen availability, unbalances in redox equilibrium and genomic instability in a framework of relentless proliferation. Chaperone induction associates with cancer progression, resistance to chemotherapy and poor prognosis. As a corollary, chaperone-targeting drugs have raised great hopes as promising anti-tumor tools [1].

The HSP90 chaperone family is particularly relevant in neoplastic growth. The two cytosolic HSP90 isoforms, HSP90α and HSP90β, interact with more than 400 proteins, termed clients (https://www.picard.ch/downloads/Hsp90interactors.pdf) and can tune the activity of several biochemical pathways whose deregulation is crucially involved in tumorigenesis. HSP90 chaperone activity facilitates conformational rearrangements in client proteins, thus avoiding their proteasomal degradation, and can be modulated by complex post-translational modifications and assembly of specific co-chaperones, allowing a high degree of plasticity to HSP90 functions [2].

Less is known about functions and clients of TRAP1, the mitochondrial chaperone of the HSP90 family. Induction of TRAP1 correlates with progression, metastasis and disease recurrence in several neoplastic models [3]. TRAP1 shields tumor cells from oxidative stress and contributes to their metabolic rewiring by down-regulating the activity of succinate dehydrogenase (SDH) and of cytochrome oxidase, the complex II and IV of the respiratory chain, respectively [4, 5].

Nonetheless, a detailed comprehension of the roles played by these chaperones in the tumorigenic process is lacking, which makes extremely difficult to reconcile under a unitary model the effects observed following their inhibition, and to design efficient therapeutic approaches. For instance, in several tumor types compounds targeting HSP90 can elicit growth arrest, but tumors start growing again after drug removal. Moreover, other HSPs, such as HSP70, can undergo compensatory induction, thus ablating inhibitor efficacy, and inhibition of any of the plethora of biological functions regulated by HSP90 can cause adverse effects in patients. In the case of TRAP1, its anti-oxidant and metabolic effects must be considered in the context of the multifaceted effects that ROS and bioenergetic adaptations could play on tumor growth. So, while in certain tumors high levels of TRAP1 are induced from very early stages of neoplastic progression in order to protect cells from oxidants [6], in other conditions oxidative stress might favor genetic instability and tumor aggressiveness. In these settings, the anti-oxidant effect of TRAP1 could hamper neoplastic progression, and indeed TRAP1 expression levels inversely correlate with tumor grade in specific tumor types [5].

Vartholomaiou, Madon-Simon *et al.* [7] add other elements to this scenario. By using a mouse model of breast cancer that exploits the oncogene polyoma virus middle T-antigen, they observe subtle and complex changes in tumorigenesis of animals where either HSP90α or TRAP1 have been knocked-out. None of these two chaperones influences tumor number or morphology, but in the absence of HSP90α tumor burden is decreased and lung metastases are less and smaller, whereas the lack of TRAP1 delays tumor onset without affecting burden or metastases. Therefore, HSP90α seems to be important in tumor growth, and TRAP1 in tumor onset. It is difficult to reconcile these differential effects caused by the absence of each chaperone to specific biological processes, as, at least *in vitro,* cells from these tumors lacking either HSP90α or TRAP1 are similarly less capable of proliferation, migration and invasion than their wild-type counterparts. Importantly, a meta-analysis carried out on a repository of gene expression data from breast cancer patients clearly indicates that mRNA expression levels of both HSP90α and TRAP1 correlate with tumor grade and, at least in the case of HSP90α, with time of survival. Accordingly, in the mouse model of Vartholomaiou *et al.* levels of the two chaperones, mainly of HSP90α, increase in tumors with respect to surrounding, non-transformed tissues.

These data highlight the need of additional information allowing to dissect how HSP90 and TRAP1 contribute to tumor growth. We must exactly understand what are the biological processes required for breast tumorigenesis in which these chaperones play a crucial role. To this aim, it is mandatory to single out relevant clients whose functions are essential for tumor onset, primary growth, invasion and metastasis. It is possible that a dynamic landscape of chaperone interactions changes according to signals funneled by oncogenic signalling pathways to HSP90 and TRAP1 via post-translational modifications (PTMs). PTMs affecting HSP90 are well established [2], even if their precise roles in neoplastic progression are far from clear. Notably, it is emerging that also TRAP1 interacts with kinases located in mitochondria, such as Src [5] and ERK1/2 [8], and that PTMs of TRAP1 are relevant for tumorigenesis. In mitochondria of cells endowed with oncogenic induction of the Ras/ERK signalling pathway, ERK1/2 phosphorylates TRAP1 and this enhances TRAP1-dependent inhibition of SDH, contributing to neoplastic growth via accumulation of the oncometabolite succinate [8].

Anti-cancer strategies aimed at targeting these molecular chaperones will greatly benefit from a detailed understanding of their mode of action and regulation in different tumor stages and types. Such information will make possible to design molecules that inhibit chaperone interactions with specific clients. These compounds could be associated with other anti-tumor drugs, with the aim of selectively targeting biological routines in which HSP90 or TRAP1 play a crucial role. As a result, neoplastic cells would receive a much stronger and selective stress stimulus, hopefully resulting in a marked improvement in the therapeutic outcome with respect to single therapies.
